# Antiprotozoal and Antiglycation Activities of Sesquiterpene Coumarins from *Ferula narthex* Exudate

**DOI:** 10.3390/molecules21101287

**Published:** 2016-09-26

**Authors:** Adnan Amin, Emmy Tuenter, Paul Cos, Louis Maes, Vassiliki Exarchou, Sandra Apers, Luc Pieters

**Affiliations:** 1 Natural Products & Food Research and Analysis (NatuRA), Department of Pharmaceutical Sciences, University of Antwerp, Universiteitsplein 1, 2610 Antwerp, Belgium; dani_amin79@yahoo.com (A.A.); emmy.tuenter@uatwerpen.be (E.T.); vasiliki.exarchou@uantwerpen.be (V.E.); sandra.apers@uantwerpen.be (S.A.); 2 Laboratory of Parasitology, Microbiology and Hygiene (LMPH), Faculty of Pharmaceutical, Biomedical and Veterinary Sciences, University of Antwerp, Universiteitsplein 1, 2610 Antwerp, Belgium; paul.cos@uantwerpen.be (P.C.); louis.maes@uantwerpen.be (L.M.)

**Keywords:** *Ferula narthex*, exudate, Apiaceae, sesquiterpene coumarins, antiprotozoal activity, antiglycation activity

## Abstract

The exudate of *Ferula narthex* Boiss. (Apiaceae) is widely used in the Indian subcontinent as a spice and because of its health effects. Six sesquiterpene coumarins have been isolated from this exudate: feselol, ligupersin A, asacoumarin A, 8′-*O*-acetyl-asacoumarin A, 10′*R*-karatavacinol and 10′*R*-acetyl-karatavacinol. Based on its use in infectious and diabetic conditions, the isolated constituents were evaluated for antimicrobial and antiglycation activities. Some compounds showed activity against protozoal parasites, asacoumarin A being the most active one against *Plasmodium falciparum* K1 (IC_50_ 1.3 μM). With regard to antiglycation activity, in the BSA-glucose test, ligupersin A displayed the highest activity (IC_50_ 0.41 mM), being more active than the positive control aminiguanidine (IC_50_ 1.75 mM). In the BSA-MGO assay, the highest activity was shown by 8′-*O*-acetyl-asacoumarin A (IC_50_ 1.03 mM), being less active than aminoguanidine (IC_50_ 0.15 mM). Hence, the antiglycation activity of the isolated constituents was due to both oxidative and non-oxidative modes of inhibition.

## 1. Introduction

The genus *Ferula* (Apiaceae) is quite diverse and includes around 180 species worldwide. It is commonly found at high altitudes in central Asia and the Middle East [1]. *Ferula narthex* Boiss. is a perennial herb, the milky exudate of which is used as spice and because of its beneficial effects on a number of ailments including cough, asthma, toothache, gastric problems, against constipation and angina pectoris [2,3,4,5]. Extracts of the herb of *F. narthex* have been reported as analgesic, insecticidal, antimicrobial and antidiabetic [6,7,8]. The genus *Ferula* is mainly known for the occurrence of coumarins, sesquiterpenes and sesquiterpene coumarins [9,10,11,12,13,14]. The extensive usage as a spice of the exudate of *F. narthex* provoked us to investigate its phytochemical composition. Based on some of the health effects listed above, extracts and isolated constituents were evaluated for antimicrobial and antiglycation activities, i.e., their ability to inhibit the formation of Advanced Glycation Endproducts (AGEs), which play a role in diabetes and its complications.

## 2. Results and Discussion

### 2.1. Structure Elucidation

Structures of the isolated compounds from *F. narthex* exudate were elucidated using ^1^H- and ^13^C-NMR (including DEPT-135 and DEPT-90) as well as 2D-NMR (COSY, HSQC and HMBC) spectroscopy (all assignments and spectra are attached as Supplementary Materials). The molecular ion was derived from the mass spectra obtained with the semi-preparative HPLC-DAD-MS system, and the UV absorption maxima from the diode-array detection.

**Figure 1:**
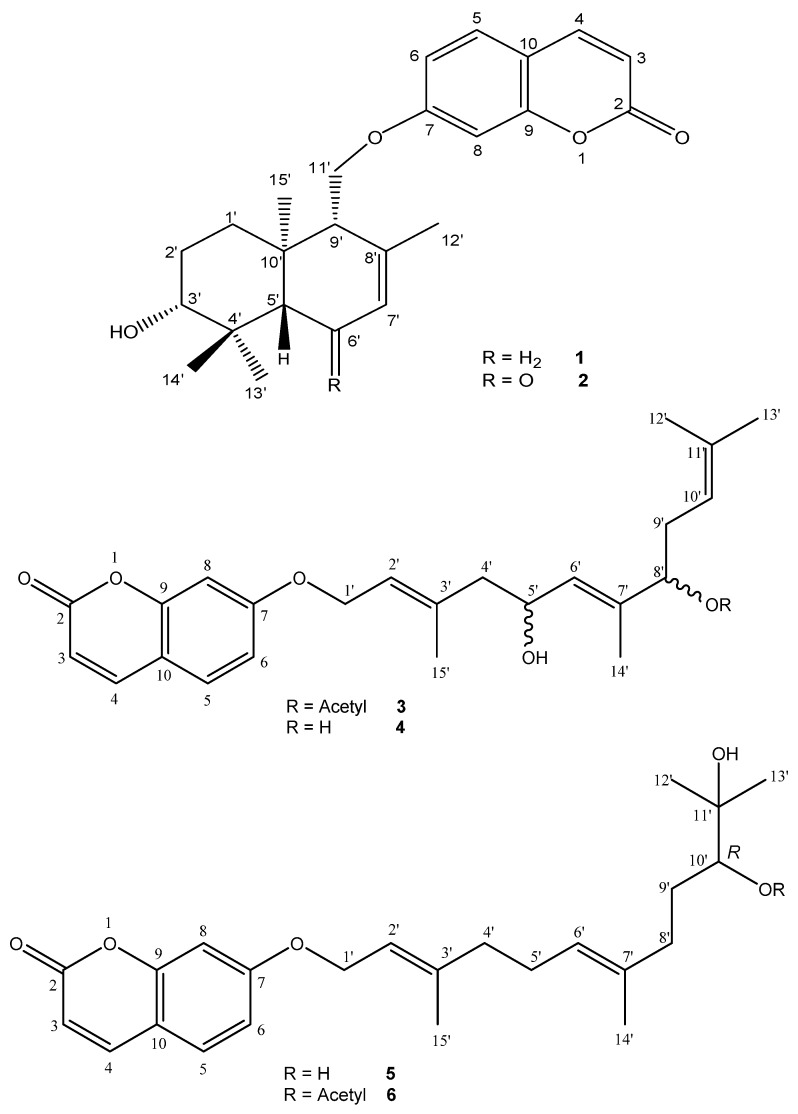
Structures of isolated constituents (**1**–**6**).

The ^1^H- and ^13^C-NMR spectra of compounds **1**–**6** (Figure 1) all showed the typical signals of a coumarin moiety, e.g., for compound **2** in ^1^H-NMR two coupled doublets at 6.24 and 7.88 ppm (*J* = 9.5 Hz, H-3 and H-4), and an aromatic 3-spin system at 7.84 (d, *J* = 8.5 Hz, H-5), 6.95 (dd, *J* = 8.5 and 2.5 Hz, H-6) and 6.98 (d, *J* = 2.5 Hz, H-8) ppm, indicating an 1,3,4-trisubstitution, were observed. In ^13^C-NMR the most typical signals of the coumarin moiety included the carbonyl group at 163.3 ppm (C-2), and the double bond signals at 114.0 and 145.9 ppm (C-3 and C-4). Similarly, in all compounds the remaining signals were due to a terpene moiety: a linear sesquiterpenyl side chain for compounds **3**–**6**, and a cyclic sesquiterpene for compounds **1** and **2**; i.e., all compounds could be classified as sesquiterpene coumarins. By comparison with published data for related products, compound **1** could readily be identified as feselol, which had been reported before from other *Ferula* species such as *F. gummosa* and *F. sinaica* [14,15]. Compound **2** was closely related, but it contained an additional carbonyl signal at 201.9 ppm (C-6′). More detailed analysis revealed that compound **2** was ligupersin A, which has previously been isolated from various medicinal plants including *Ligularia persica* [16], *F. gummosa* and *F. flabelliloba* [11,12]. Both compounds **1** and **2** showed a doublet of doublets in ^1^H-NMR for H-3′ (*J* = 10.9/4.5 Hz and 11.0/4.3 Hz, respectively), which is indicative for an equatorial position of the hydroxy group at C-3′ [16]. According to Saidkhodzhaev and Malikov [17], the sesquiterpene moieties of fesolol and ligupersin A have the same relative configuration, being different only in the presence or absence of a carbonyl functionality at C-6′. Conferol is the C-3′ epimer of feselol [17]. Su et al. have reported fesolol (**1**) as well as conferol from *F. pallida* [18]. However, the reported NMR assignments are not in agreement with the equatorial position of the C-3′ hydroxy group in feselol, and its axial position in conferol, suggesting that both compounds may have been confused. This is also apparent from the ^13^C-NMR assignment of the methyl groups at position C-4′, which occur around 28–29 ppm and 15–16 ppm when the C-3′ OH is equatorial as in ligupersin A and feselol (**1**), but around 28 ppm and 22 ppm when it is axial as in ferocaulidin, the C-3′ epimer of ligupersin A (**2**) [16]. Since Bashir et al. [19] refer to Su et al. [18] to report conferol from *F. narthex*, there is some doubt whether conferol or rather feselol is concerned. In addition, they have reported some other coumarin derivatives from whole plant material of *F. narthex*, i.e., fnarthexone, fnarthexol, conferone and umbelliferone.

In the series of the coumarins substituted with a linear sesquiterpene side chain, compound **4** could be identified as asacoumarin A, or 5′,8′-dihydroxy-umbelliprenin. This compound has been isolated before from *F. assa-foetida* and *F. foetida* [20,21]. Compound **3** was the 8′-*O*-acetyl derivative of compound **4**, or 8′-acetoxy-5′-hydroxy-umbelliprenin (8′-*O*-acetyl-asacoumarin A). This compound has previously been reported from asafetida, the gum resin obtained by incision of the roots of various plants from the genus *Ferula* [9,22] The absolute configuration at C-5′ and C-8′ of asacoumarin A (**4**) and 8′-*O*-acetyl-asacoumarin A (**3**) has not been reported yet, but the specific optical rotation of the compounds isolated here was similar to published values, indicating identical configurations. Finally, compound **5** was identified as 10′*R*-karatavacinol, isolated before from *F. assa-foetida* and *F. sinaica*; [21,22,23] and compound **6** was the 10′-acetyl derivative of compound **5**, or 10′*R*-acetoxy-11′-hydroxy-umbelliprenin (10*R*′-acetyl-karatavacinol). This compound has previously been isolated from *F. assa-foetida* [22]. Configurational assignments of **5** and **6** were made by comparison of the specific optical rotation with published values.

### 2.2. Antimicrobial Activity

Because of its traditional use, the crude extract and fractions of the *F. narthex* exudate were evaluated for antibacterial and antifungal activities. Highest levels of antimicrobial and antifungal activities were observed for the methanol fraction, i.e., an IC_50_ of 19.9 μg/mL against *Staphylococcus aureus* and 22.0 μg/mL against *Microsporum canis*, whereas this fraction was also found to be cytotoxic towards MRC-5 cells (IC_50_ 36.5 μg/mL) (Table 1). Similarly, the *n*-hexane fraction was active (IC_50_ 8.3 μg/mL) against *M. canis,* whereas the chloroform fraction was cytotoxic (IC_50_ 49.8 μg/mL) against MRC-5 cells. The isolated compounds **1**, **3**–**5** were evaluated in an integrated screening panel for antimicrobial activity (compounds **2** and **6** were not tested due to the limited amount available). Although the methanol fraction showed activity in the preliminary screening, none of the tested compounds presented antibacterial (*S. aureus*, *Escherichia coli*) or antifungal activities (*Candida albicans*, *Aspergillus fumigatus*) in the test range. It may be hypothesized that the individual compounds may act synergistically in the crude fractions that were found active. A few investigations have reported antibacterial properties of sesquiterpene coumarins [24,25]. Since the latter compounds were structurally quite different from the ones isolated here, this may be due to specific structure-activity relationships.

However, promising activities against some protozoal parasites were observed (Table 2). Feselol (**1**) was moderately active against *Plasmodium falciparum* K1 (IC_50_ 22.4 μM), whereas equal levels of inhibition were observed for *Trypanosoma brucei* (IC_50_ 8.1 μM), *Trypanosoma cruzi* (IC_50_ 8.6 μM) and *Leishmania infantum* (IC_50_ 6.8 μM), accompanied however by cytotoxicity on peritoneal murine macrophages (PMM) (IC_50_ 8.0 μM). Antileishmanial activity against the promastigote stage (IC value of 11.51 μg/mL) has been reported for conferol [19]. The linear sesquiterpene coumarins **3**, **4** and **5** were also active in the antiprotozoal assays. Asacoumarin A (**4**) was found most active against *P. falciparum* K1 (IC_50_ 1.3 μM), while less pronounced activities were observed against *T. brucei* (IC_50_ 32.6 μM), *T. cruzi* (IC_50_ 10.5 μM) and *L. infantum* (IC_50_ 12.7 μM), accompanied however by PMM cytotoxicity (IC_50_ 32.0 μM). 8′-*O*-Acetyl-asacoumarin A (**3**) also displayed promising activity against *P. falciparum* K1 (IC_50_ 7.4 μM), and less pronounced activities against *T. brucei* (IC_50_ 32.4 μM), *T. cruzi* (IC_50_ 19.1 μM) and *L. infantum* (IC_50_ 12.7 μM), again accompanied by PMM cytotoxicity (IC_50_ 32.0 μM). Similarly, 10*′R*-karatavacinol (**5**) presented activity against *P. falciparum* K1 (IC_50_ 16.0 μM), *T. brucei* (IC_50_ 32.4 μM), *T. cruzi* (IC_50_ 9.4 μM) and *L. infantum* (IC_50_ 32.4 μM), again accompanied by PMM cytotoxicity (CC_50_ 32.0 μM). All tested compounds were cytotoxic against MRC-5 cells in various degrees. In this way, cytotoxicities could be evaluated in a primary cell (PMM) as well as in a cell line (MRC-5), and were in agreement with each other, compound **1** being the most cytotoxic. In vitro antileishmanial activity has been reported before for 7-prenyloxy-coumarins from *Ferula szowitsiana* [26]. Umbelliprenin obtained from the same species was found to inhibit the growth of human M4Beu metastatic pigmented malignant melanoma cells through cell-cycle arrest in phase G1 and induction of caspase-dependent apoptosis [27,28].

### 2.3. Antiglycation Activity

All isolated compounds (**1**–**6**) were tested for their antiglycation potential in the BSA-Glucose and BSA-MGO models. Glucose-mediated protein glycation models are generally used to determine inhibition of the formation of Advanced Glycation Endproducts (AGEs). AGEs are involved in many degenerative diseases, such as diabetes and its complications, cardiovascular and neurodegenerative diseases, and the physiological process of ageing. However, AGE-protein adducts can be formed both under oxidative and non-oxidative conditions, and therefore AGEs inhibitors should be differentiated from common antioxidants [29]. Evaluation of the inhibition of AGEs formation starting from glucose and BSA involves all possible mechanisms. However, Reactive Carbonyl Substances (RCS) such as glyoxal and methyl glyoxal that are intermediate products of the Maillard reaction, have already undergone the oxidation processes starting from glucose. Inhibition of their reaction with BSA is representative of non-oxidative glycation reactions.

Primarily in the BSA-glucose test, ligupersin A (**2**) displayed the highest activity (IC_50_ 0.41 mM), being more active than the positive control aminiguanidine, followed by asacoumarin A (**4**) (IC_50_ 1.83 mM), whereas compounds **1** (47% inhibition at the highest test concentration of 2 mM), **3** (44% inhibition), **5** (32% inhibition) and **6** (36% inhibition) did not reach the 50% inhibition level (Table 3). On the other hand, in the BSA-MGO assay, the highest activity was shown by 8′-*O*-acetyl-asacoumarin A (**3**) (IC_50_ 1.03 mM), followed by **1** (IC_50_ 1.71 mM) and **5** (IC_50_ 1.83 mM), whereas the 50% inhibition level was not reached for compounds **2** (40% inhibition at the highest test concentration of 2 mM), **4** (35% inhibition) and **6** (20% inhibition). Therefore, it could be concluded that the antiglycation potential of the isolated constituents was due to both oxidative and non-oxidative modes of inhibition.

## 3. Materials and Methods 

### 3.1. General Experimetal Procedures

All solvents were analytical grade and obtained from Fisher Scientific (Leicestershire, UK) and Acros Organics (Geel, Belgium). All chemicals and reagents were purchased from Acros Organics or Sigma-Aldrich (St. Louis, MO, USA). The solvents for HPLC were purchased from Fisher Scientific. RiOS water was prepared by reverse osmosis and water for HPLC was dispensed by a Milli-Q system, both from Millipore (Bedford, MA, USA). Water was passed through a 0.22 μm membrane filter before usage.

Analytical thin layer chromatography (TLC) was carried out on silica gel 60 F_254_ plates (20 × 20 cm) (Merck, Darmstadt, Germany). The spraying reagent *p*-anisaldehyde was prepared by mixing 0.5 mL *p*-anisaldehyde (Sigma-Aldrich) with 10 mL glacial acetic acid, 85 mL methanol and 5 mL sulfuric acid.

Flash column chromatography was performed on a Reveleris iES system from Grace (Columbia, MD, USA) using the Reveleris^®^ Navigator ™ software. The system was equipped with a binary pump with four solvent selection, an ultraviolet (UV) and evaporative light scattering detector (ELSD) and a fraction collector. A pre-packed Flash Grace Reveleris silica cartridge (80 g) with a particle size of 40 μm was used. The ELSD carrier solvent was isopropyl alcohol.

HPLC analysis was carried out on an Agilent 1200 series system with degasser, quaternary pump, automatic injection, thermostatic column compartment and a diode array detector (DAD) (Agilent Technologies, Santa Clara, CA, USA). A silica based Gracesmart C_18_ column (250 × 4.6 mm, 5 μm) (Grace) and Luna C_18_ column (250 × 4.6 mm, 5 μm) (Phenomenex, Torrence, CA, USA) were used, together with a suitable precolumn to endure the lifetime of columns. 

Isolation of compounds was carried out using a Luna C_18_ column (250 × 10.0 mm, 5 μm) (Phenomenex) on a semi-preparative HPLC system with DAD and ESIMS detectors. This system included a sample manager, injector and collector (2767), a quaternary gradient module (2545), a System Fluidics Organizer, an HPLC-pump (515), a photodiode array detector (2998) and a Micromass Quattro mass spectrometer with TQD, all supplied by Waters (Milford, MA, USA). MassLynx version 4.1 (Waters) was used to process the data.

NMR spectra were recorded on a Bruker DRX-400 instrument (Rheinstetten, Germany), operating at 400 MHz for ^1^H and at 100 MHz for ^13^C, employing a 3-mm broadband inverse (BBI) probe or a 5-mm dual ^1^H/^13^C probe using standard Bruker pulse sequences. DEPT-135, DEPT-90 and two-dimensional NMR (COSY, HSQC, HMBC) experiments were recorded. In order to assist structure elucidation, a ^13^C-NMR library was used (NMR Predict version 4.8.57, Modgraph, Oaklands Welwyn, Herts AL6 ORJ, UK). Deuterated solvents including CDCl_3_ (99.8% D), CD_3_OD (99.8% D) and CD_3_CN (99.8% D) were purchased from Sigma-Aldrich [30].

The specific optical rotation was determined on a Jasco P-2000 polarimeter (Easton, MD, USA). The samples were dissolved in methanol and optical rotation was recorded at 589 nm with a path length of 50 mm.

### 3.2. Plant Material

*Ferula narthex* exudate was collected in September 2012 from the district Chitral (KPK), Pakistan. The samples were identified at the Islamabad Herbarium in the Taxonomy Department, Quaid-I-Azam University, Islamabad, Pakistan, where the voucher specimen was deposited (voucher No. 568 BC, accession no. ISL-72568). The semisolid exudate was stored below 20 °C until further use. 

### 3.3. Extraction and Isolation

Dried plant exudate (1 kg) was extracted with 90% (*v*/*v*) methanol by double cold maceration. The extract was filtered through Whatman No.1 filter paper using a vacuum pump. The collected filtrate was dried using a rotary evaporator under reduced pressure below 40 °C. The resultant semisolid material was lyophilized with a final yield of 87.1 g, and stored below 20 °C. Liquid–liquid partitioning was performed on the crude extract according to a standard extraction scheme (Supplementary Materials, Scheme S1). After partitioning with different solvents as shown in the scheme, *n*-hexane (1.21 g), methanol 90% (25.0 g), chloroform (1.96 g), ethyl acetate (0.2 g), *n*-butanol (4.87 g) and aqueous fractions (53.86 g) were obtained. All collected fractions were dried under reduced pressure at 40 °C, lyophilized and stored below 20 °C.

Normal phase TLC for all obtained fractions was executed using various solvent systems as mobile phase. The solvent systems used included CH_2_Cl_2_/MeOH (65:35) with a few drops of NH_4_OH for the methanol fraction; CH_2_Cl_2_/MeOH (72:28) for the chloroform fraction; *n*-hexane/CH_2_Cl_2_ (93:7) for the *n*-hexane fraction; CH_2_Cl_2_/MeOH (40:60) for the *n*-butanol fraction and CH_2_Cl_2_/MeOH (15:85) for the aqueous fraction. Developed TLC plates were examined under UV at 254 nm and 366 nm and after spraying with *p*-anisaldehyde reagent.

An aliquot of 0.8 g from the methanol 90% fraction was dissolved in 2 mL methanol and mixed with 1.1 g silica; the mixture was dried with nitrogen gas. The dried extract was loaded on a prepacked Flash Grace Reveleris^®^ silica cartridge of 80 g. The compounds were eluted using a gradient from methylene chloride to methanol. Based on UV and ELSD detection, multiple subfractions were collected. All fractions were further analyzed by TLC and similar fractions were combined. In this way 16 subfractions were obtained. Flash chromatography with similar conditions was repeated when necessary. Finally, based on TLC profiling, subfractions FME1 (200 mg), FME2 (150 mg), FME4 (220 mg) and FME5 (210 mg) were selected for further HPLC profiling.

Likewise, an aliquot of 0.8 g from the chloroform fraction was loaded on a flash column as discussed above. The compounds were eluted using a gradient from methylene chloride to methanol. Based on UV and ELSD detection different subfractions were collected. All fractions were analyzed by TLC and similar fractions were combined as described above; in this way five subfractions were obtained. Based on TLC profiling subfractions FCL1 (120 mg), FCL2 (140 mg), FCL3 (180 mg), FCL4 (100 mg) and FCL5 (140 mg) were selected for further HPLC analysis.

Similarly, an aliquot of 0.8 g from the *n*-butanol fraction was subjected to flash chromatography with a gradient from methylene chloride to methanol as previously explained. Finally, 10 subfractions were obtained. As noticed for the chloroform fraction, the TLC profiling of the *n*-butanol fraction shared some common bands with the methanol and chloroform fractions. Based on TLC analysis, subfractions FB2 (140 mg), FB4 (125 mg), FB5 (154 mg), FB6 (200 mg) and FB7 (116 mg) were selected for HPLC analysis. 

The methanol fraction and all obtained subfractions FME1, FME2, FME4 and FME5 were analyzed by HPLC using an optimized acetonitrile/H_2_O + 0.1% formic acid gradient, ranging from 20% acetonitrile to 80% in 60 min at a flow rate of 1 mL/min. Samples were prepared in a concentration range from 1–10 mg/mL in methanol. The isolation of pure compounds was performed by semi-preparative HPLC-DAD-MS using the same gradient at 3 mL/min, yielding compounds **1** (5.5 mg), **2** (4.6 mg) and **3** (6.2 mg).

Similarly, the crude chloroform fraction and subfractions FCL1, FCL2, FCL3, FCL4 and FCL5 were analyzed by HPLC using an optimized acetonitrile/H_2_O + 0.1% formic acid gradient ranging from 35% acetonitrile to 80% in 45 min at a flow rate of 1 mL/min. The compounds were isolated by semi-preparative HPLC-DAD-MS using the same gradient at a flow rate of 3 mL/min. Finally compounds **2** (2.5 mg), **3** (4.2 mg) and **5** (4.3 mg) were isolated.

The crude *n*-butanol fraction and subfractions FB2, FB4, FB5, FB6 and FB7 were analyzed by HPLC using an optimized acetonitrile/H_2_O + 0.1% formic acid gradient ranging from 40% acetonitrile to 65% in 50 min at a flow rate of 1 mL/min. Compound isolation was performed by semi-preparative HPLC-DAD-MS using the same gradient as HPLC at a flow rate of 3 mL/min. Compounds **2** (3.6 mg), **5** (4.2 mg) and **6** (4.8 mg) were finally isolated.

*Feselol* (**1**). ${\left[\alpha \right]}_{D}^{20}$ −21.7 (*c* 0.0067, MeOH); UV (acetonitrile/H_2_O) λ_max_ 243, 325 nm; ^1^H and ^13^C-NMR: Supplementary Materials, Table S1; ESI-MS (positive ion mode): *m*/*z* 405 [M + Na]^+^ consistent with a molecular formula C_24_H_30_O_4_.

*Ligupersin A* (**2**). ${\left[\alpha \right]}_{D}^{20}$ −56.2 (*c* 0.45, MeOH); UV (acetonitrile/H_2_O) λ_max_ 218, 236, 296, 325 nm; ^1^H- and ^13^C-NMR: Supplementary Materials, Table S2; ESI-MS (positive ion mode): *m*/*z* 419 [M + Na]^+^ consistent with a molecular formula C_24_H_28_O_5_.

*8′-O-Acetyl-asacoumarin A* (**3**). ${\left[\alpha \right]}_{D}^{20}$ +3.3 (*c* 0.0048, MeOH); UV (acetonitrile/H_2_O) λ_max_ 205, 325 nm; ^1^H- and ^13^C-NMR: Supplementary Materials, Table S3; ESI-MS (positive ion mode): *m*/*z* 463 consistent with a molecular formula C_26_H_32_O_6_.

*Asacoumarin A* (**4**). ${\left[\alpha \right]}_{D}^{20}$ +4.2 (*c* 0.0058, MeOH); UV (acetonitrile/H_2_O) λ_max_ 203, 325 nm; ^1^H- and ^13^C-NMR: Supplementary Materials, Table S4; ESI-MS (positive ion mode): *m*/*z* 421 [M + Na]^+^ consistent with a molecular formula C_24_H_30_O_5_.

*10′R*-*Karatavacinol* (**5**). ${\left[\alpha \right]}_{D}^{20}$ +7.4 (*c* 0.0041, MeOH); UV (acetonitrile/H_2_O) λ_max_ 211, 318 nm; ^1^H- and ^13^C-NMR: Supplementary Materials, Table S5; ESI-MS (positive ion mode): *m*/*z* 423 [M + Na]^+^ consistent with a molecular formula C_24_H_32_O_5_.

*10*′*R*-*Acetyl-karatavacinol* (**6**). ${\left[\alpha \right]}_{D}^{20}$ +13.5 (*c* 0.0048, MeOH); UV (acetonitrile/H_2_O) λ_max_ 213, 321 nm; ^1^H- and ^13^C-NMR: Supplementary Materials, Table S6; ESI-MS (positive ion mode): *m*/*z* 465 [M + Na]^+^ consistent with a molecular formula C_26_H_34_O_6_.

### 3.4. Antimicrobial Activity

Fractions and isolated compounds were evaluated for antimicrobial activity in an integrated screening panel as reported before [31,32,33,34].

### 3.5. Antiglycation Activity

The BSA (bovine serum albumin) -glucose and BSA-MGO (methyl glyoxal) antiglycation assays were carried out as reported before [31]. The test protocol is attached as Supplementary Materials.

## 4. Conclusions

Based on findings of the current investigation, it could be hypothesized that the isolated sesquiterpene coumarins may contribute in part to the health effects of the exudate of *F. narthex*, used as a spice, in diabetic conditions and in antimicrobial applications. Toxicity of a particular chemotype of *Ferula communis* has been attributed to *C*-prenylated coumarins such as ferulenol and ferprenine [35]. However, it concerns 4-hydroxy-coumarins prenylated in position 3. Since this type of terpenyl coumarins has not been found in the present work in the *F. narthex* exudate, this implies that such substances do not pose any safety concern with regard to its human consumption.

## Figures and Tables

**Table 1:** Antibacterial, antifungal and cytotoxic activities of *Ferula narthex* fractions.

Sample	IC_50_ μg/mL				
	*S. aureus*	*E. coli*	*C. albicans*	*M. canis*	MRC-5
MeOH 90%	19.9	>64	>64	22.0	36.5
Chloroform	>64	>64	>64	>64	49.8
Ethyl acetate	>64	>64	>64	>64	>64
*n*-Hexane	>64	>64	>64	**8.3**	>64
*n*-Butanol	>64	>64	>64	>64	>64
Aqueous	>64	>64	>64	>64	>64

MRC-5: Human fetal lung fibroblasts; *S. aureus*: *Staphylococcus aureus*; *E. coli*: *Escherichia coli*; *C. albicans*: *Candida albicans*. Reference compounds: Tamoxifen (MRC-5) IC_50_ 11.4 μg/mL; erythromycin (*S. aureus*) IC_50_ 11.2 μg/mL; trimethoprim (*E. coli*) IC_50_ 0.25 μg/mL; miconazole (*C. albicans*) IC_50_ 5.99 μg/mL; terbinafine (*M. canis*) IC_50_ 0.11 μg/mL.

**Table 2:** Antiprotozoal and cytotoxic activities of isolated compounds from *Ferula narthex* (IC_50_, μM).

Compound No.	IC_50_ (μM)					
	MRC5	Pf-K1	*T. bruc.*	*T. cruz.*	*L. inf.*	PMM
**1**	8.0	22.4	8.1	8.6	6.8	8
**3**	31.7	7.4	32.4	19.1	12.7	32
**4**	11.7	1.3	32.6	10.5	12.7	32
**5**	20.4	16.0	32.4	9.4	32.4	32

MRC-5: Human fetal lung fibroblasts; Pf-K1: Plasmodium falciparum K1; *T. bruc.*: *Trypanosoma brucei*; *T. cruz.*: *Trypanosoma cruzi*; *L .inf.*: *Leishmania infantum*; PMM: Peritoneal Murine Macrophages. Reference compounds: Tamoxifen (MRC-5), IC_50_ 11.3 μM; suramine (*T. brucei*), IC_50_ 0.03 μM; fungizone (*L. inf.*), IC_50_ 1.1 μM; chloroquine (Pf-K1), IC_50_ 0.16 μM; benznidazole (*T. cruzi*), IC_50_ 3.3 μM; erythromycin (*S. aureus*), IC_50_ 11.3 μM; chloramphenicol (*E. coli*), IC_50_ 4.9 μM; miconazole (*C. albicans*), IC_50_ 10.5 μM; terbinafine (*A. fumigatus*), IC_50_ 0.8 μM.

**Table 3:** Antiglycation (AGEs) activity of compounds **1**–**6**.

Compound No.	BSA-Glucose BSA-MGO			
	% Inhibition ^a^	IC_50_ (mM)	% Inhibition ^b^	IC_50_ (mM)
**1**	47	-		1.71
**2**		0.41	40	-
**3**	44	-		1.03
**4**		1.83	35	-
**5**	32	-	-	1.86
**6**	36	-	20	-
Aminoguanidine		1.75		0.15
Quercetin		0.23		0.35

^a^ at 0.25 mM; ^b^ at 2 mM.

## Supplementary Materials

Supplementary materials can be accessed at: http://www.mdpi.com/1420-3049/21/10/1287/s1.
