# Supplementary material for: Antiprotozoal and Antiglycation Activities of Sesquiterpene Coumarins from Ferula narthex Exudate
Source: Molecules. 2016 Sep 26;21(10):1287. doi: 10.3390/molecules21101287 (PMC6274357; doi:10.3390/molecules21101287)
Supplement: Supplementary file 1 [file molecules-21-01287-s001.pdf]

Supplementary Materials: Antiprotozoal and Antiglycation Activities of Sesquiterpene Coumarins from *Ferula narthex* Exudate

Adnan Amin, Emmy Tuenter, Paul Cos, Louis Maes, Vassiliki Exarchou, Sandra Apers and Luc Pieters

1. Antiglycation Assay (BSA-Glucose Assay)

The antiglycation assay was performed according to the method developed by Matsuura et al. (2002) [1] with minor modifications. The reaction mixture (300 µL) contained bovine serum albumin (BSA) (10 mg/mL, 135 µL), D-glucose (500 mM, 135 µL) dissolved in phosphate buffer (50 mM, pH 7.4, containing sodium azide (0.02%, added to prevent bacterial growth) and test compounds (30 µL), at different final concentrations (1.5–0.023 mM for isolated constituents or 100–1.17 µg/mL for extracts) dissolved in 100 % DMSO. The mixtures were incubated at 60 °C for 48 h. After incubation, the reaction mixture was allowed to cool down at room temperature. Then 100 µL reaction mixture was transferred to a new plastic tube (1.5 mL) and the reaction was stopped by adding 10 µL of 100% (*w*/*v*) trichloroacetic acid (TCA) and to precipitate proteins. The TCA-added mixture was kept at 4 °C for 10 min, followed by centrifugation (14,000 rpm, 4 °C, 4 min). The supernatant containing unreacted D-glucose, test sample and interfering substances was discarded, whereas the precipitate containing AGEs-BSA was redissolved with 0.8 mL alkaline PBS (137 mM NaCl, 8.1 mM Na_2HPO_4, 2.68 mM KCl, 1.47 mM KH_2PO_4, pH 10). The change in fluorescence intensity (excitation 335 nm, emission 385 nm; excitation 370, emission 440) due to formation of AGEs was monitored by spectrofluorometry (Tecan™ Infinite M200, Giessen, The Netherlands). In order to eliminate interference by autofluorescence of the test compounds, a parallel incubation of test substance with BSA at 60 °C without D-glucose was performed for all samples. Quercetin (0.5–0.0312 mM final concentration) and aminoguanidine (3–0.046 mM final concentration) were used as reference compounds.

2. The AGEs Inhibition was Calculated as

$$\% \text{ inhibition} = \{1 - (\text{FBSA} + \text{glucose} + \text{test substance} - \text{FBSA} + \text{test substance})/(\text{FBSA} + \text{glucose} - \text{FBSA})\} \times 100 \quad (1)$$

where F is the fluorescence intensity. The concentration required for 50% inhibition (IC_{50}) was calculated using using Sigma plot® 13.0.

3. Protein-Glyoxal Interaction (BSA-MGO Assay)

The antiglycation assay using methylgloxyl (MGO) was performed according to the method developed by Peng et al. (2007) [2] with slight modifications. Briefly, methylglyoxal (135 µL, 5.75 mM) was incubated with BSA (135 µL, 10 mg/mL) dissolved in phosphate buffer (50 mM, pH 7.4, containing sodium azide (0.02%), added to prevent bacterial growth) and test compounds (30 µL) at different final concentrations (1.5–0.023 mM) in 100% DMSO. The reaction mixture was incubated at 37 °C for one week. Control solutions only contained methylglyoxal (135 µL, 5.75 mM) BSA (135 µL, 1 0 mg/mL) and DMSO (100%) only. The blank samples of similar composition were also prepared simultaneously and kept at 4 °C for one week. The change in fluorescence intensity (excitation 335 nm, emission 385 nm; excitation 370, emission 440) due to formation of AGEs was monitored by spectrofluorometry (Tecan® Infinite M200, Giessen, The Netherlands). Aminoguanidine (3–0.046 mM final concentration) was used as reference compound. The AGEs inhibition was calculated as

$$\% \text{ AGEs inhibition} = [1 - (S - Sb)/(C - Cb)] \times 100 \quad (2)$$

where S and C were fluorescence of test samples (in DMSO) and control (test mixtures containing only DMSO) incubated at 37 °C, and where Sb and Cb were fluorescence for samples incubated at 4 °C. The concentration required for 50% inhibition (IC_{50}) was calculated using using Sigma plot® 13.0.

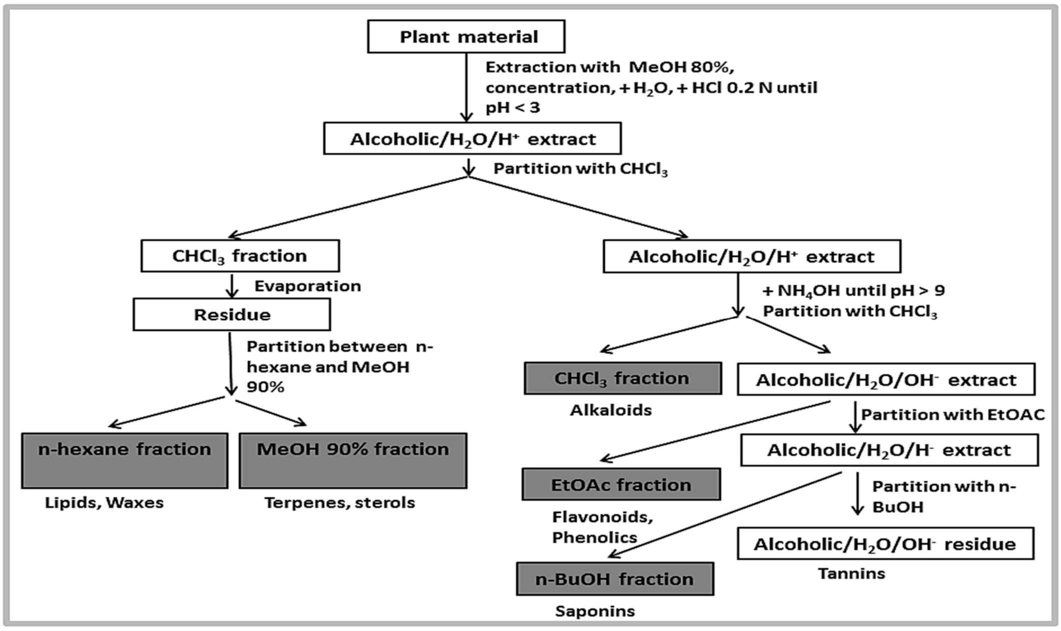

Scheme S1. Liquid-liquid extraction scheme.

Table S1. ^{1}H- and ^{13}C-NMR assignments of feselol (**1**) recorded in MeOH-d_4

Position	δ_H (ppm); Multiplicity; *J* (Hz)	δ_C (ppm)
1		
2		163.4
3	6.23; d; *J* = 9.5	113.6
4	7.87; d; *J* = 9.5	145.9
5	7.52; d; *J* = 8.5	130.5
6	6.91; br d; *J* = 8.5	114.4
7		163.7
8	6.93; br s	102.2
9		157.2
10		114.1
1′	2.07; m	24.3
2′	1.63; 0.92; m	28.0
3′	3.23; dd; *J* = 10.9; 4.5	79.6
4′		39.9
5′	1.26; m	50.9
6′	2.0; m	39.0
7′	5.53, br s	124.5
8′		133.9
9′	2.20, br s	55.4
10′		37.0
11′	4.08; dd; *J* = 10; 3.4<br>4.24; dd; *J* = 10; 5.8	68.2
12′	1.69; s	21.8
13′	0.87; s	15.3
14′	0.98; s	28.7
15′	0.93; s	15.9

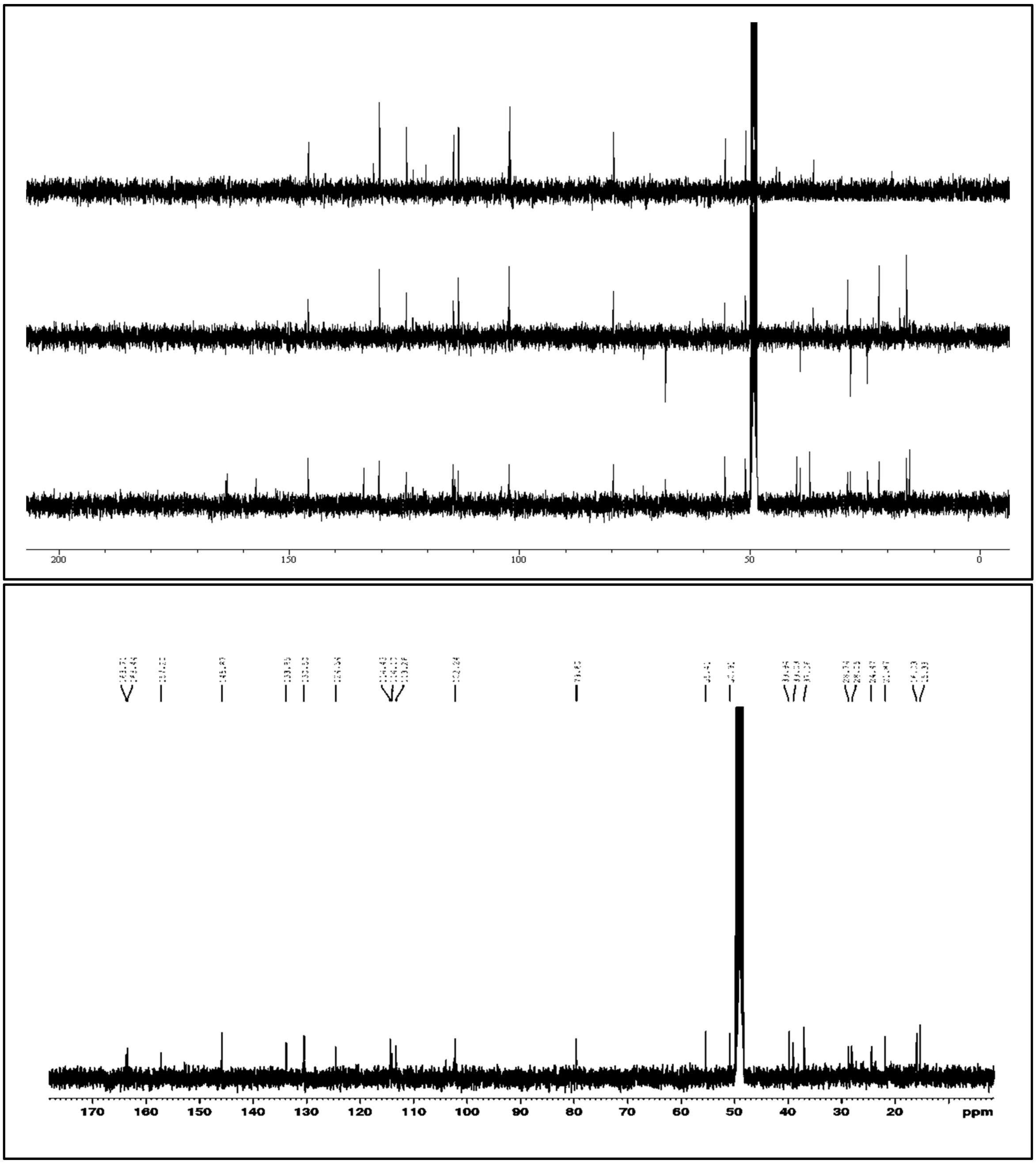

Figure S1. ^{13}C-NMR, DEPT-135 and DEPT-90 spectra of feselol (**1**) recorded in MeOH-d_4.

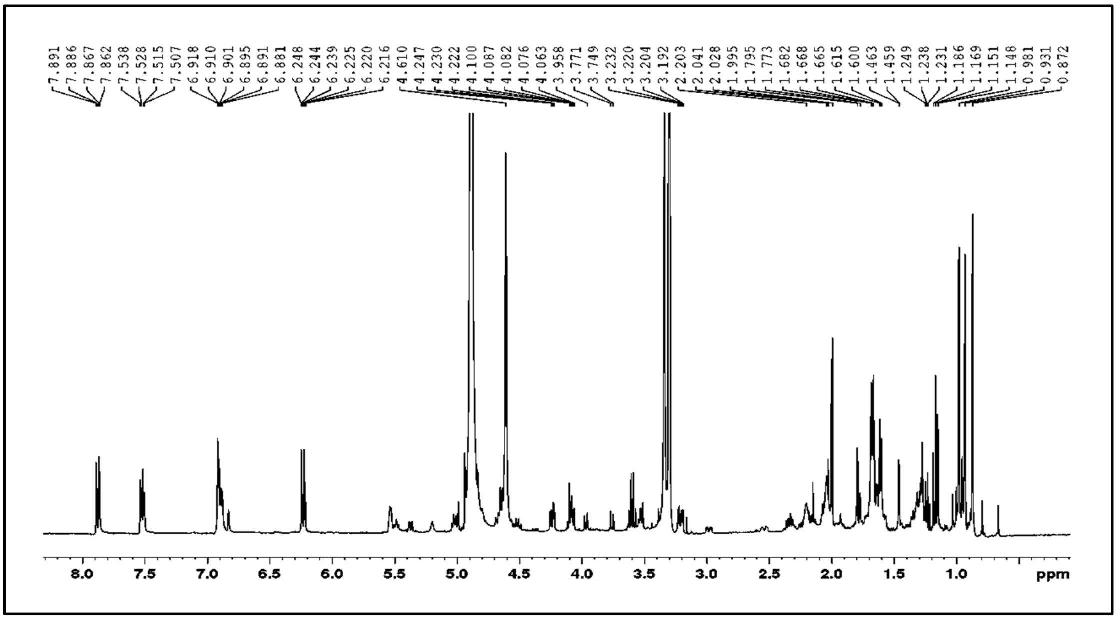

Figure S2. ^{1}H-NMR spectrum of feselol (**1**) recorded in MeOH-d_4.

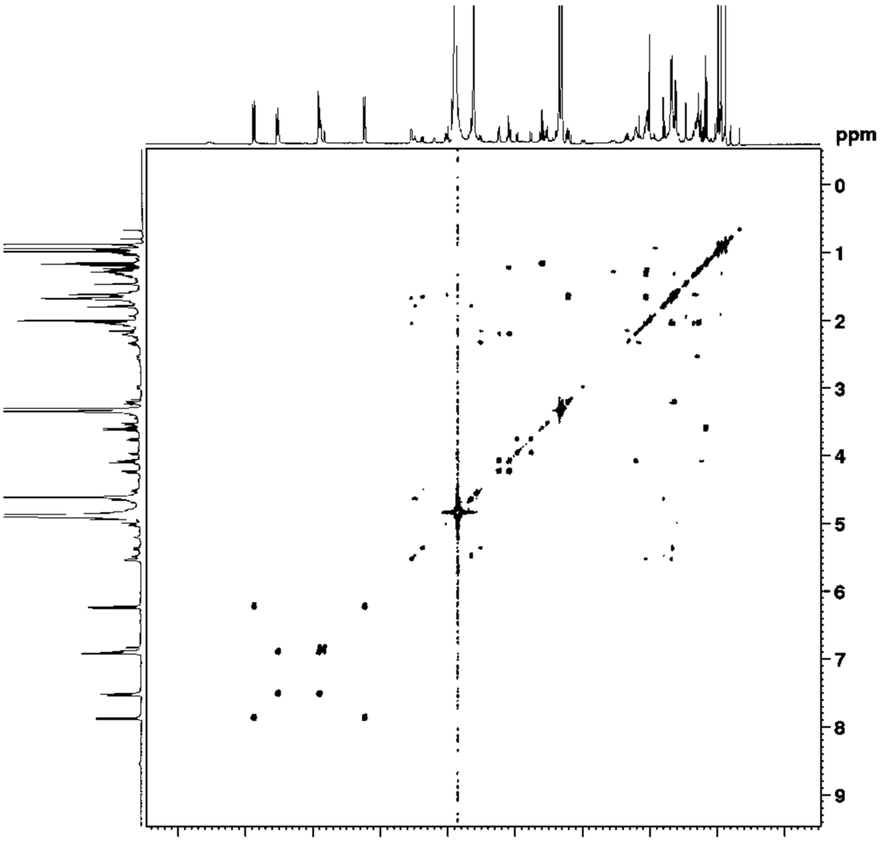

Figure S3. COSY spectrum of feselol (**1**) recorded in MeOH-d_4.

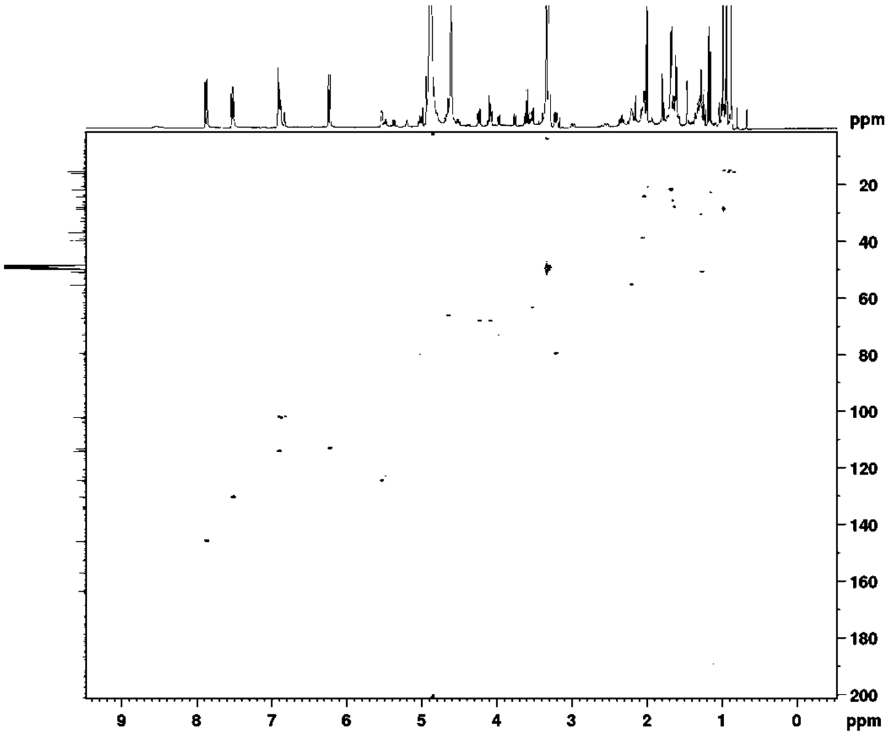

Figure S4. HSQC spectrum of feselol (**1**) recorded in MeOH-d_4.

Table S2. ^{1}H- and ^{13}C-NMR assignments of ligupersin A (**2**) recorded in MeOH-d_4.

Position	δ_H (ppm); multiplicity; *J* (Hz)	δ_C(ppm)
2		163.3
3	6.24; dd; *J* = 9.5	114.0
4	7.88; d; *J* = 9.5	145.9
5	7.84; d; *J* = 8.5	130.7
6	6.95; *J* = 8.5; 2.5	114.5
7		163.5
8	6.98; d; *J* = 2.5	102.6
9		159.6
10		114.4
1′	1.67; 2.06; m	38.5
2′	1.65; 1.72; m	27.6
3′	3.20; dd; *J* = 11.0; 4.3	79.4
4′		39.1
5′	2.33; s	63.7
6′		201.9
7′	5.84; br s	130.1
8′		157.4
9′	2.81; m	56.5
10′		43.6
11′	4.32; dd; *J* = 10.5, 5.2<br>4.40; dd; *J* = 10.5, 3.0	67.1
12′	2.0; s	22.0
13′	1.16; s	16.0
14′	1.26; s	29.1
15′	1.06; s	16.7

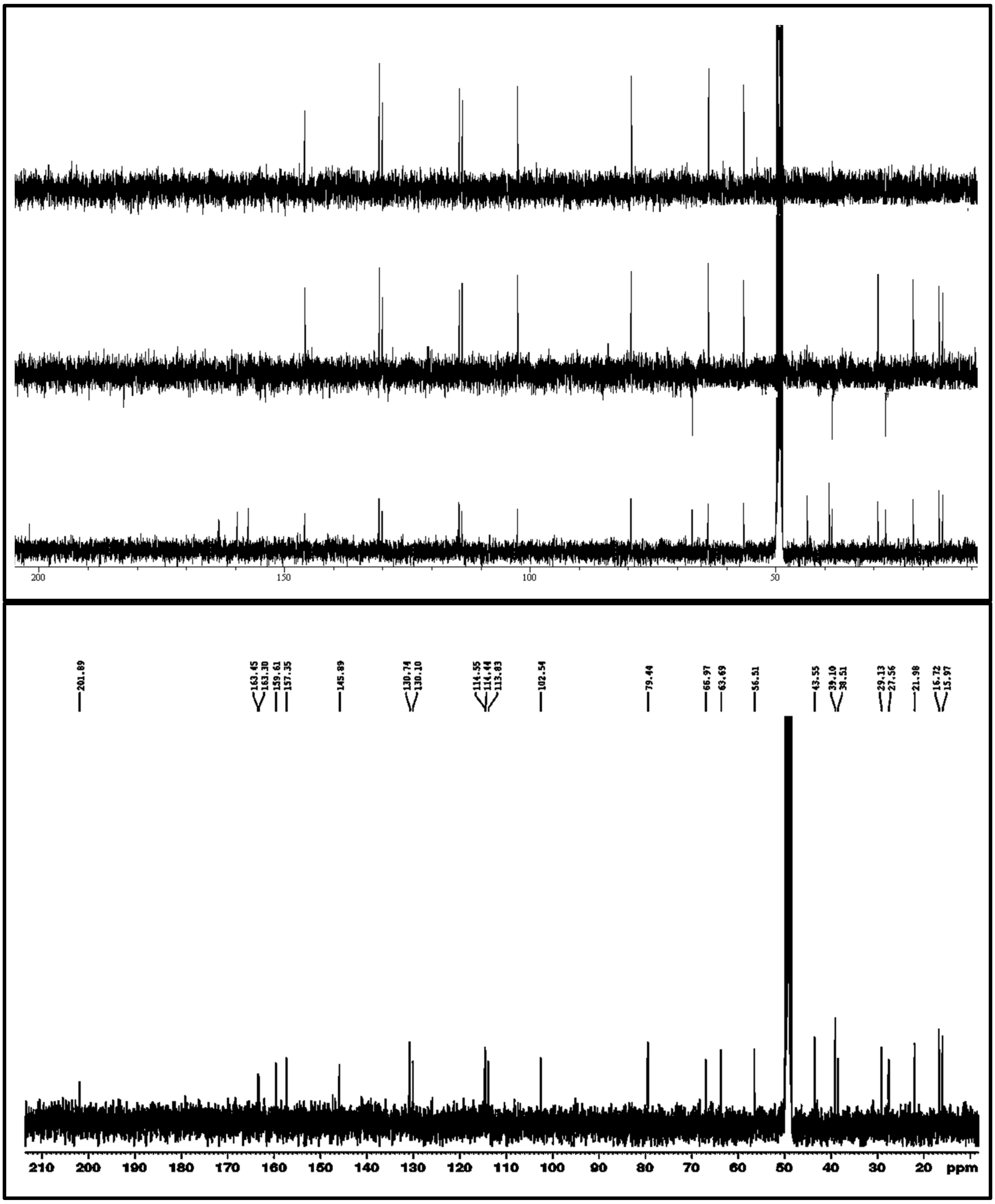

Figure S5. ^{13}C-NMR, DEPT-135 and DEPT-90 spectra of ligupersin A (**2**) recorded in MeOH-*d*$_4$.

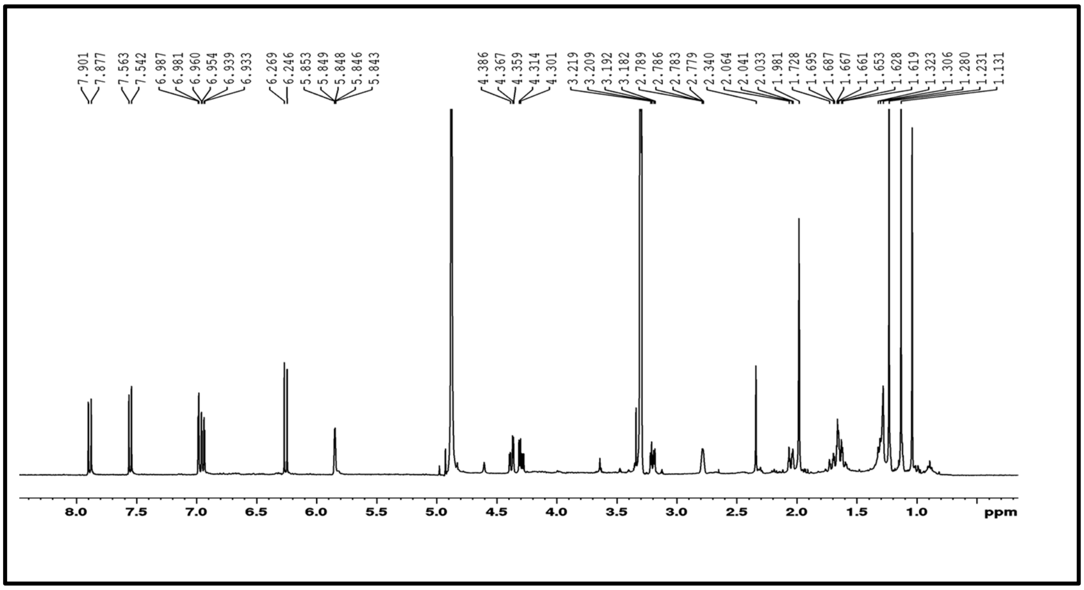

Figure S6. ^{1}H-NMR spectrum of ligupersin A (**2**) recorded in MeOH-*d*4.

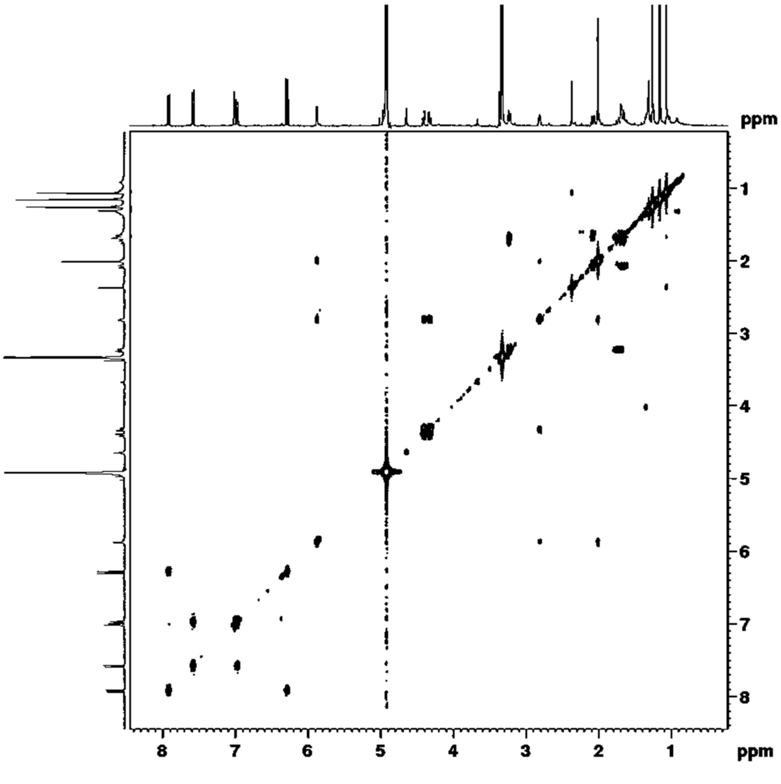

Figure S7. COSY spectrum of ligupersin A (**2**) recorded in MeOH-*d*4.

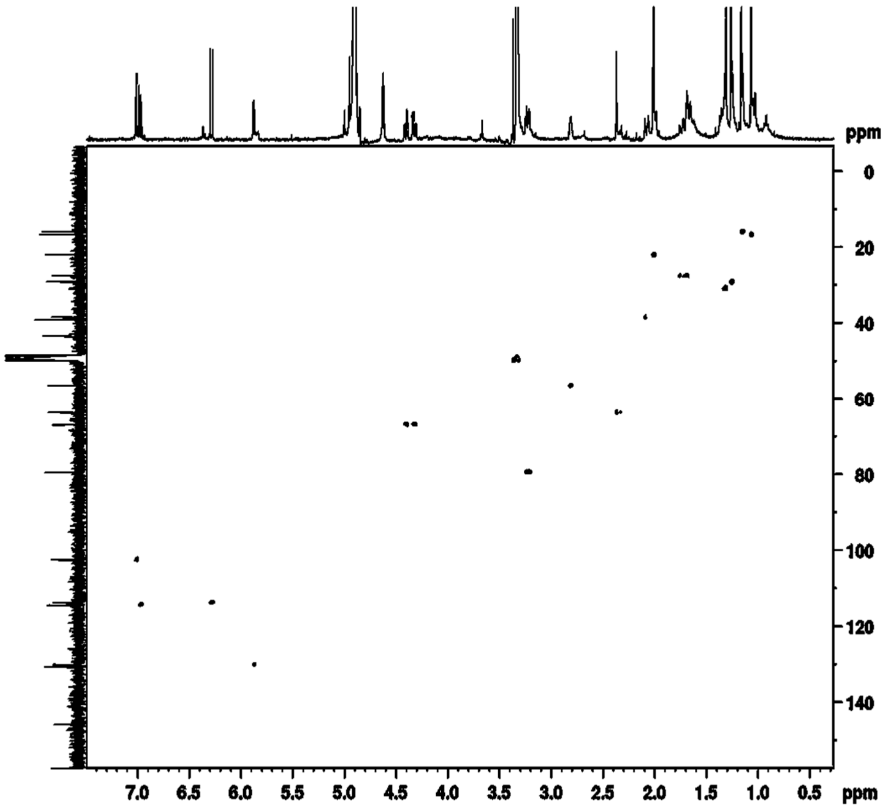

Figure S8. HSQC spectrum of ligupersin A (**2**) recorded in MeOH-*d*4.

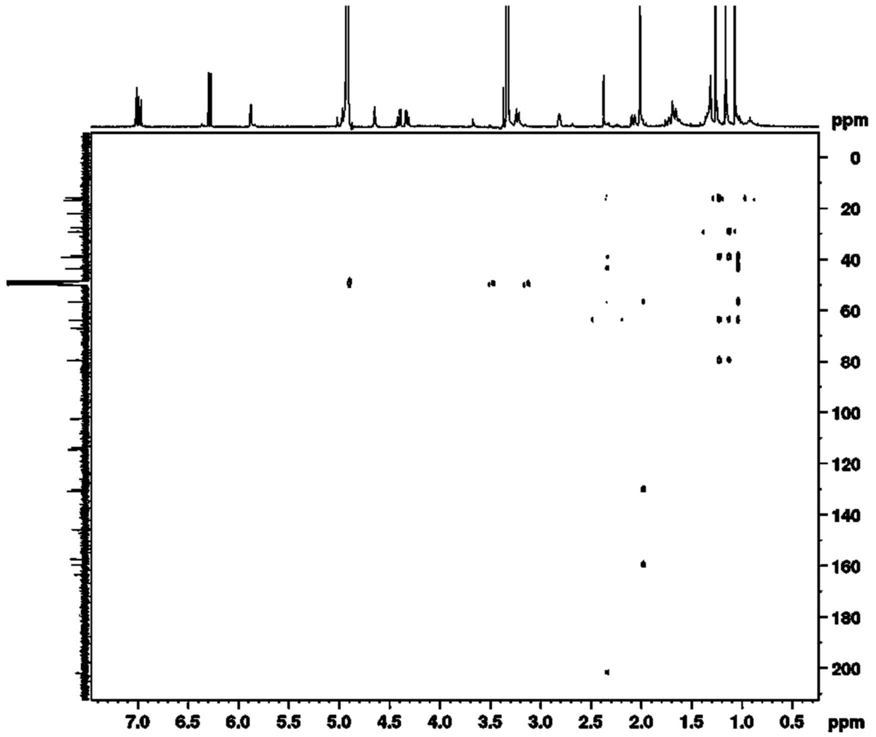

Figure S9. HMBC spectrum of ligupersin A (**2**) recorded in MeOH-*d*4.

Table S3. ^{1}H- and ^{13}C-NMR assignments of 8′-*O*-acetyl-asacoumarin A (**3**) recorded in MeOH-d_4.

Position	δH (ppm); multiplicity; *J* (Hz)	δC (ppm)
2		163.4
3	6.21; d; *J* = 8.5 Hz	113.4
4	7.90; d; *J* = 9.5 Hz	145.8
5	7.51; d; *J* = 8.5 Hz	130.5
6	6.90; dd; *J* = 2.5; 8.0 Hz	114.4
7		163.8
8	6.84; d; *J* = 2.5	102.6
9		157.1
10		114.0
1′	4.64; br d; *J*= 6.2	66.7
2′	5.50; br t; *J*=6.2	123.2
3′		139.3
4′	2.34; m<br>2.16; m	48.3
5′	4.51; m	67.3
6′	5.40; br d; *J*= 8.2	131.9
7′		136.4
8′	5.02; t; *J*= 7.0	80.0
9′	2.33; m<br>2.24; m	32.8
10′	4.83; overlapped with solvent peak	120.3
11′		135.4
12′	1.60; br s	12.8
13′	1.70; s	18.0
14′	1.68; s	17.4
15′	1.80; br s	26.0
Ac	2.00; s	21.0<br>172.0

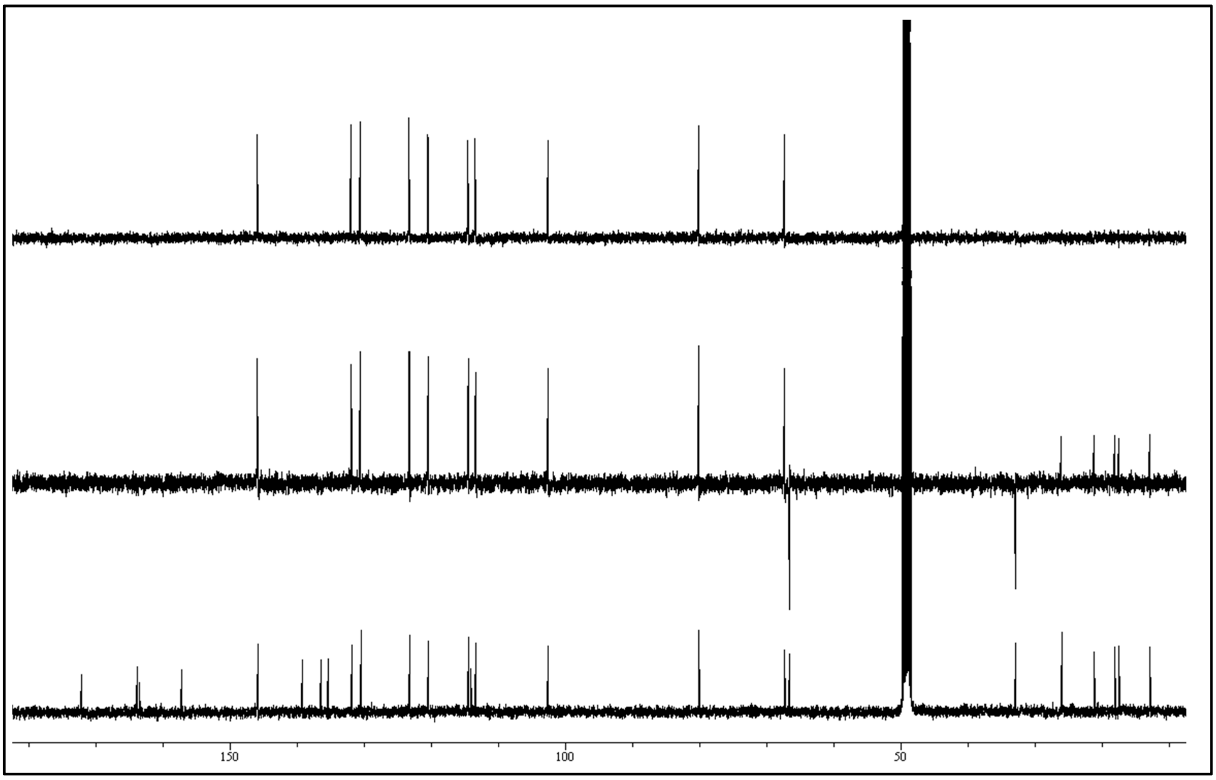

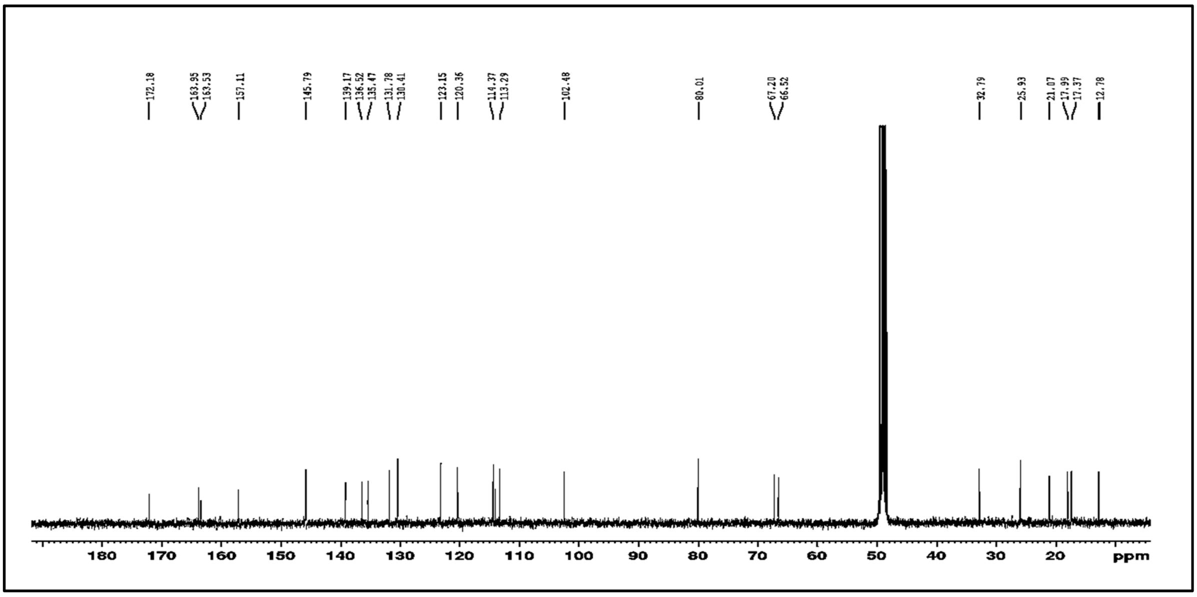

Figure S10. ^{13}C-NMR, DEPT-135 and DEPT-90 spectra of 8′-*O*-acetyl-asacoumarin A (**3**) recorded in MeOH-d_4.

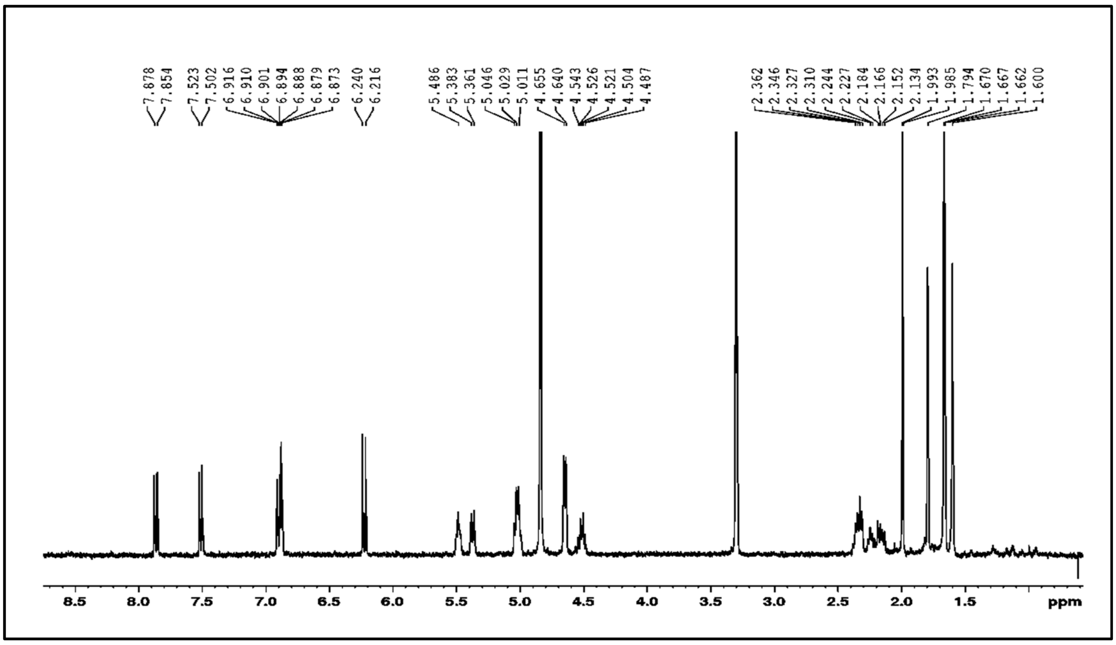

Figure S11. ^{1}H-NMR spectrum 8′-*O*-acetyl-asacoumarin A (**3**) recorded in MeOH-d_4.

Table S4. ^{1}H- and ^{13}C-NMR assignments of asacoumarin A (**4**) recorded in MeOH-d_4.

Position	δ_H (ppm); multiplicity; J (Hz)	δ_C (ppm)
2		163.4
3	6.22; d; J = 9.5	113.2
4	7.88; d; J = 9.5	145.8
5	7.50; d; J = 8.5	130.3
6	6.88; dd; J = 8.0, 2.5	114.4
7		163.8
8	6.85; d; J = 2.5	102.2
9		157.1
10		113.9
1′	4.64;d; J = 6.4	66.5
2′	5.50; br t; J = 6.2	123.0
3′		140.1
4′	2.34; m<br>2.14; m	48.3
5′	4.51; dd; J = 15.0, 6.4	67.4
6′	5.3; d; J = 8.2	130.4
7′		139.3
8′	3.89; t; J = 6.7	78.5
9′	2.11; m	34.9
10′	5.05; t; J = 7.0	121.8
11′		133.9
12′	1.60; br s	18.0
13′	1.66; s	26.0
14′	1.8; s	17.3
15′	1.60; br s	11.6

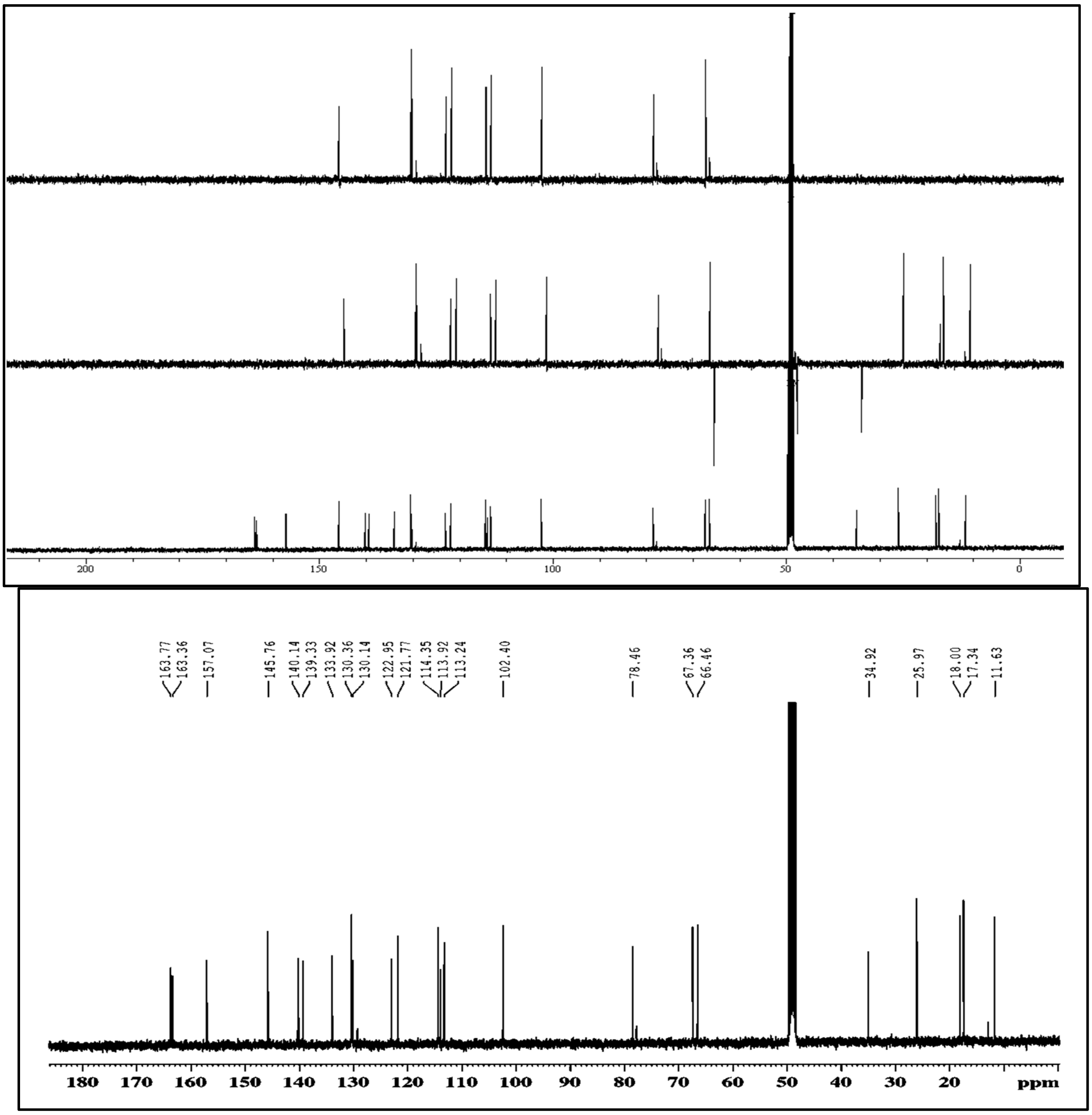

Figure S12. ^{13}C-NMR, DEPT-135 and DEPT-90 spectra of asacoumarin A (**4**) recorded in MeOH-d_4.

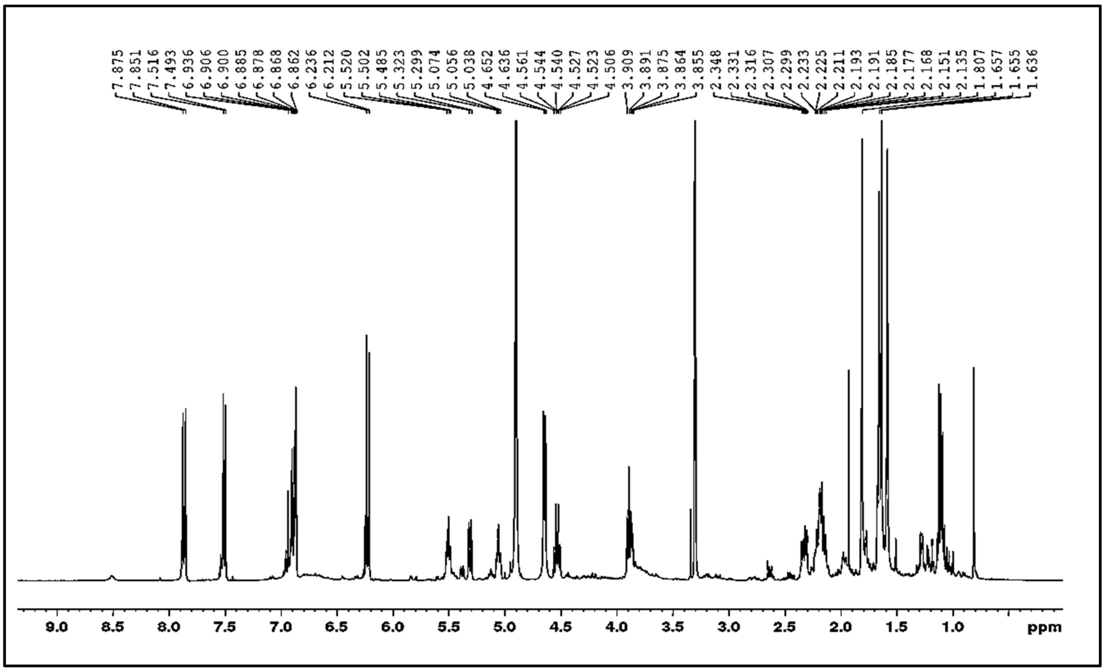

Figure S13. ^{1}H-NMR spectrum of asacoumarin A (**4**) recorded in MeOH-d_4.

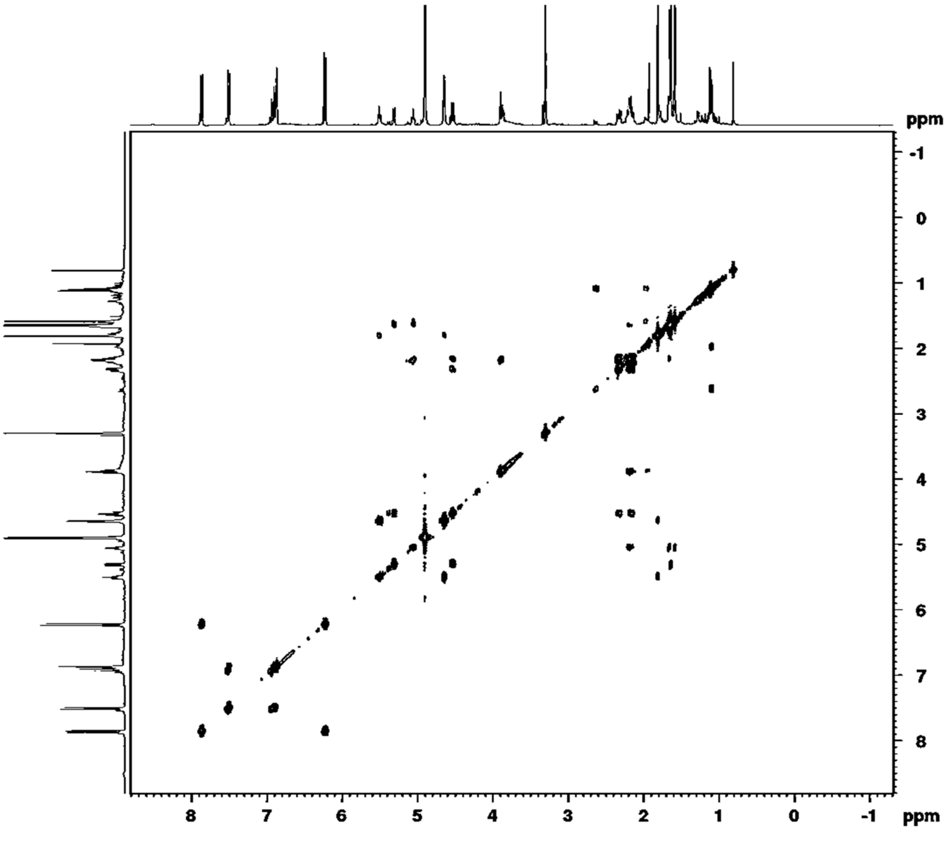

Figure S14. COSY spectrum of asacoumarin A (**4**) recorded in MeOH-d_4.

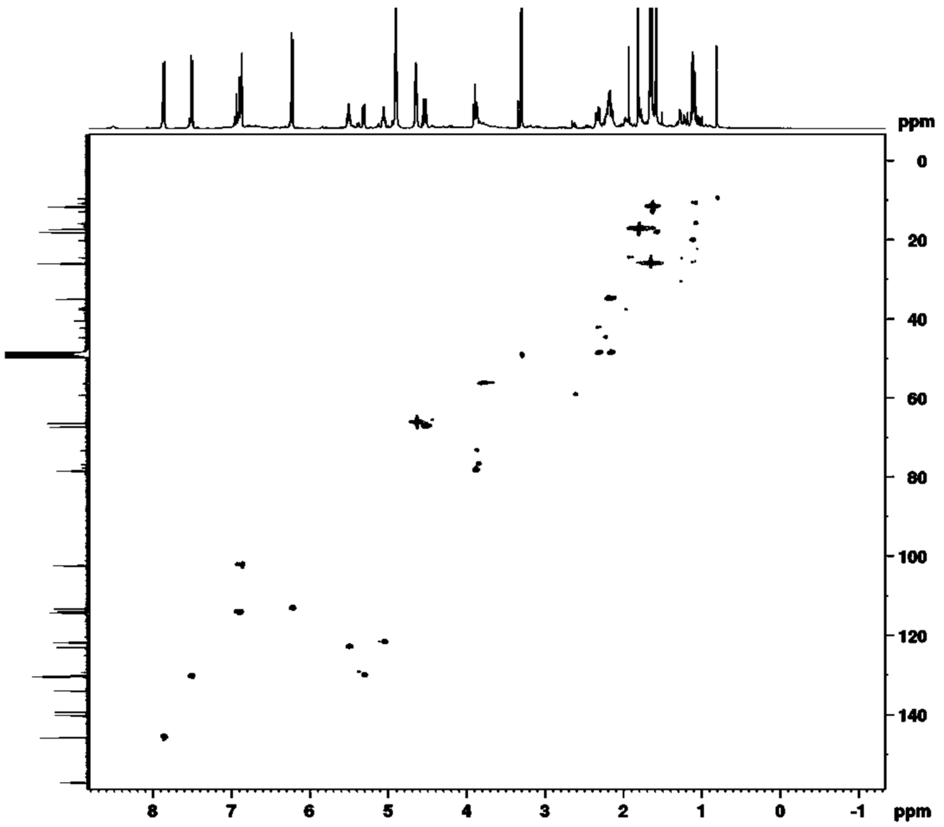

Figure S15. HSQC spectrum of asacoumarin A (**4**) recorded in MeOH-*d*4.

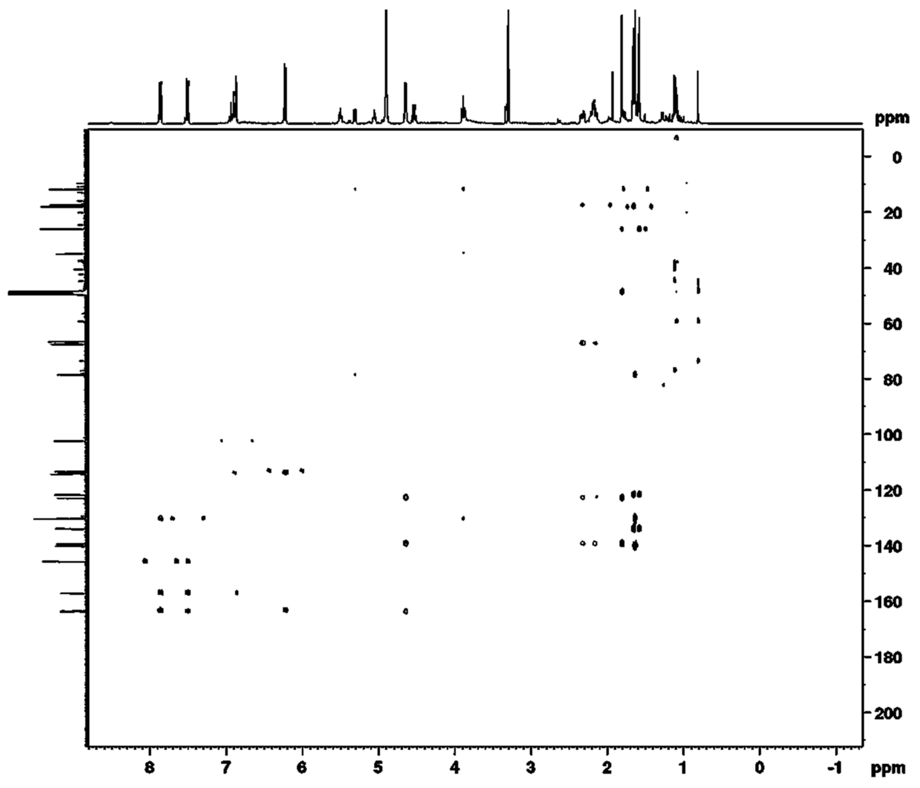

Figure S16. HMBC spectrum of asacoumarin A (**4**) recorded in MeOH-*d*4.

Table S5. ^{1}H- and ^{13}C-NMR assignments of 10′*R*-karatavacinol (**5**) recorded in MeOH-*d*$_4$.

Position	δ_H (ppm); multiplicity; *J* (Hz)	δ_C (ppm)
2		163.8
3	6.22; d; *J* = 9.5	113.8
4	7.90; d; *J* = 9.5	145.8
5	7.51; d; *J* = 8.5	130.3
6	6.91; dd; *J* = 8.0, 2.5	114.4
7		163.4
8	6.90; d; *J* = 2.5	102.5
9		157.0
10		113.2
1′	4.65; d; *J* = 6.0	66.6
2′	5.46; t; *J* = 6.0	120.3
3′		142.8
4′	2.17; m	37.8
5′	1.20; m	30.8
6′	5.16; br t; *J* = 6.0	125.0
7′		136.5
8′	1.32; m	40.5
9′	1.70; m	27.2
10′	3.21; dd; *J* = 10.0, 2.0	77.7
11′		73.7
12′	1.10; s	25.0
13′	1.13; s	16.7
14′	1.61; s	25.6
15′	1.77; s	16.2

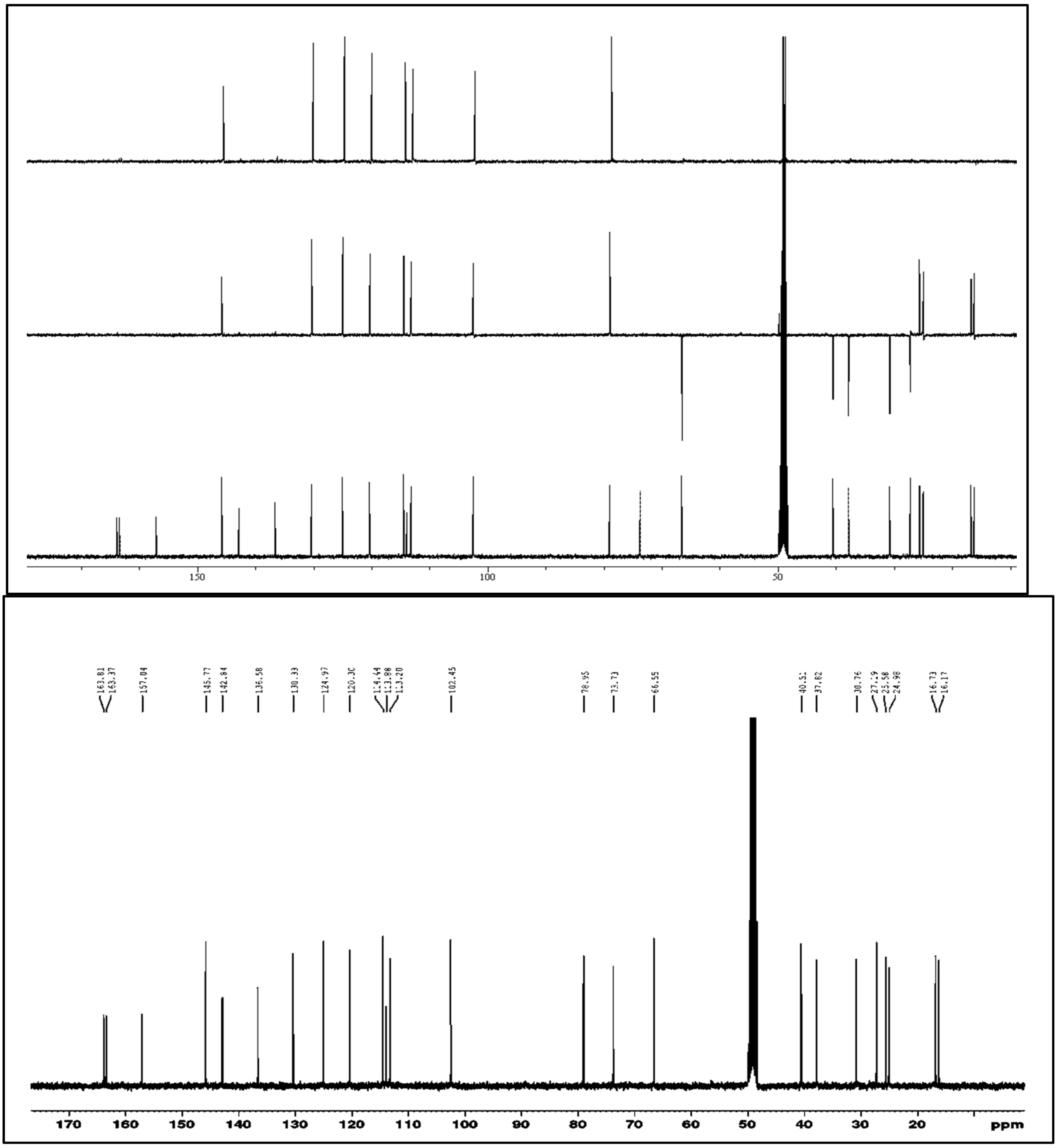

Figure S17. ^{13}C-NMR, DEPT-135 and DEPT-90 spectra of 10′*R*-karatavacinol (**5**) recorded in MeOH-d_4.

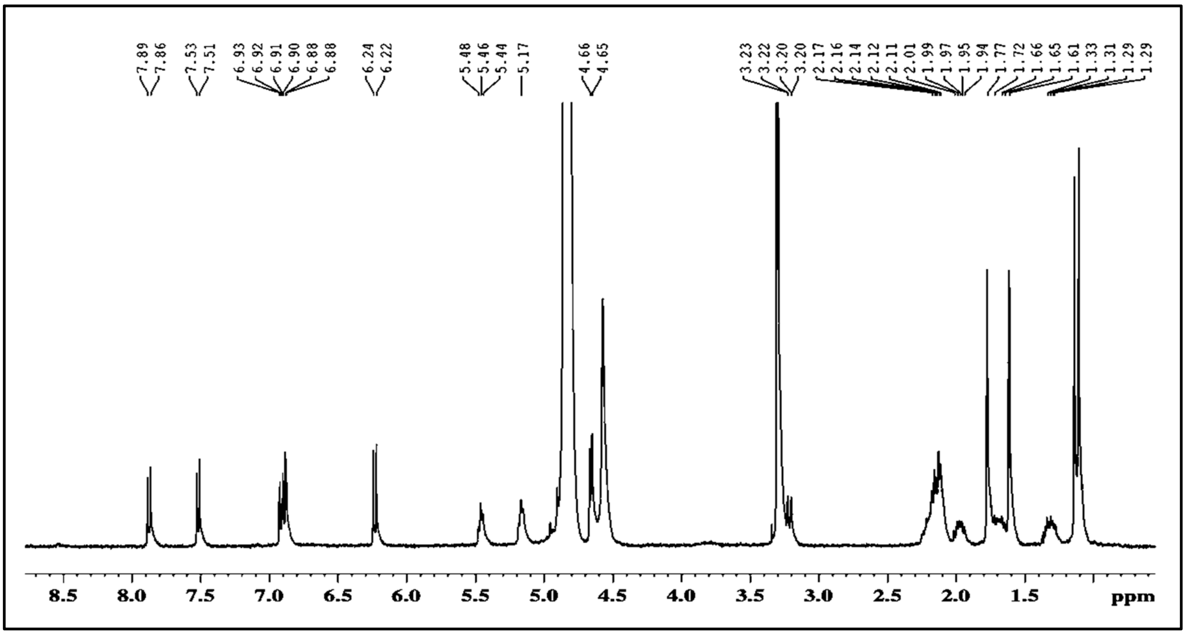

Figure S18. ^{1}H-NMR spectrum of 10′*R*-karatavacinol (**5**) recorded in MeOH-*d*$_4$.

Table S6. ^{1}H- and ^{13}C-NMR assignments of 10′*R*-acetyl- karatavacinol (**6**) recorded in MeOH-*d*$_4$.

Position	δ$_H$ (ppm); multiplicity; *J* (Hz)	δ$_C$ (ppm)
2		164.0
3	6.23; d; *J* = 9.5	113.2
4	7.90; d; *J* = 9.5	145.9
5	7.51; d; *J* = 8.5	130.4
6	6.91; dd; *J* = 8.0, 2.5	114.5
7		163.5
8	6.90; d; *J* = 2.5	102.5
9		157.1
10		113.9
1′	4.66; d; *J* = 6.0	66.6
2′	5.50; t; *J* =6.0	120.5
3′		143.0
4′	2.16–2.05; m	40.4
5′	2.16–2.05; m	27.2
6′	5.16; br t; *J* = 6.0	125.5
7′		135.7
8′	1.92; m	37.4
9′	1.67; m	29.0
10′	4.75; dd; *J* = 10.5, 2.0	80.6
11′		72.8
12′	1.12; s	25.7
13′	1.13; s	25.9
14′	1.60; s	16.0
15′	1.77; s	16.7
OAc (C-10′)	2.0; s	21.1<br>172.8

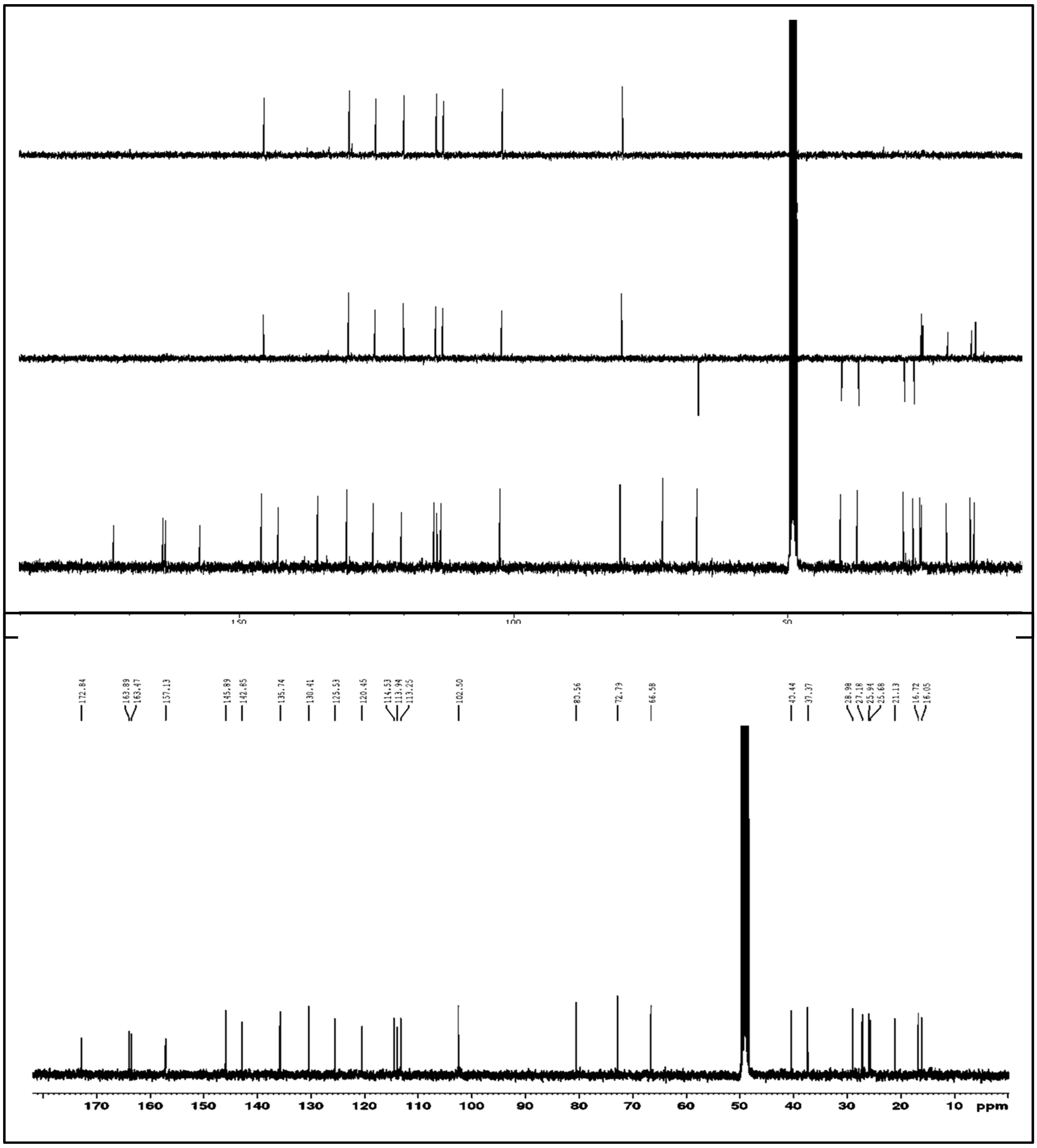

Figure S19. ^{13}C-NMR, DEPT-135 and DEPT-90 spectra of 10′*R*-acetyl-karatavacinol (**6**) recorded in MeOH-*d*$_4$.

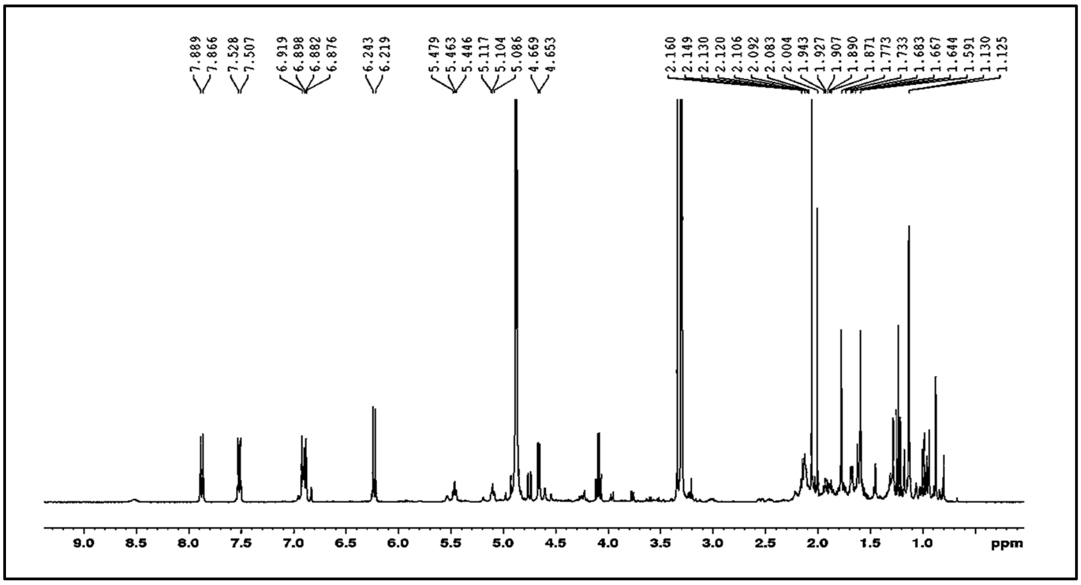

Figure S20. ^{1}H-NMR spectrum of 10′*R*-acetyl-karatavacinol (**6**) recorded in MeOH-d_4.

References

1. Matsuura, N.; Aradate, T.; Sasaki, C.; Kojima, H.; Ohara, M.; Hasegawa, J. Screening system for the Maillard reaction inhibitor from natural product extracts. *J. Health Sci. Surveillance Syst.* **2002**, *48*, 520–526.
2. Peng, X.; Zheng, Z.; Cheng, K.W.; Shan, F; Ren, G.X.; Chen, F.; Wang, M. Inhibitory effect of mungbean extract and its constituents vitexin and iso-vitexin on the formation of advanced glycation end products. *Food Chem.* **2007**, *106*, 475–481.
